# Hilbert transform‐based time‐series analysis of the circadian gene regulatory network

**DOI:** 10.1049/iet-syb.2018.5088

**Published:** 2019-08-01

**Authors:** Shiju S., K. Sriram

**Affiliations:** ^1^ Center for Computational Biology Indraprastha Institute of Information Technology Delhi New Delhi 110020 India

**Keywords:** time series, genetics, Hilbert transforms, stochastic processes, circadian rhythms, signal processing, medical signal processing, phase model, experimental time series, circadian time series, circadian rhythms, circadian gene regulatory network, deterministic time series, stochastic time series, fruit fly model, phase response curves, period sensitivity, phase locking, phase slips, Hilbert transform, time‐series analysis, signal processing

## Abstract

In this work, the authors propose the Hilbert transform (HT)‐based numerical method to analyse the time series of the circadian rhythms. They demonstrate the application of HT by taking both deterministic and stochastic time series that they get from the simulation of the fruit fly model *Drosophila melanogaster* and show how to extract the period, construct phase response curves, determine period sensitivity of the parameters to perturbations and build Arnold tongues to identify the regions of entrainment. They also derive a phase model that they numerically simulate to capture whether the circadian time series entrains to the forcing period completely (phase locking) or only partially (phase slips) or neither. They validate the phase model, and numerics with the experimental time series forced under different temperature cycles. Application of HT to the circadian time series appears to be a promising tool to extract the characteristic information about circadian rhythms.

## 1 Introduction

Signal processing tools have been used extensively to understand the properties of circadian rhythms. For example, wavelet transform was applied to the circadian experimental time series to extract period variations in the suprachiasmatic nucleus (SCN) [1, 2] and to study the interaction of ultradian oscillations with circadian rhythm [3]. While Forger [4] applied Fourier transform to the circadian model with biochemical feedback to understand the effect of feedback loops to variations in the period, Escalante‐Martnez *et al.* [5] employed Laplace transform to model circadian rhythms. Laplace transform, though suitable to analyse stationary data, cannot apply to realistic non‐stationary biological signals. Fourier transform can detect the frequency components in the signal, but, does not provide information about the instantaneous frequency. Wavelet transform can estimate the instantaneous frequency with better resolution in time but depends strongly on the selection of proper wavelets. Moreover, wavelet analyses are computationally intensive and have to take care of harmonics and edge effects [2].

Mathematical models of circadian rhythms help to understand the dynamics that govern the oscillator based on the molecular mechanism of gene expression. These mechanistic models are highly complex, non‐linear, high‐dimensional and cannot be subjected to linear signal processing tools like Fourier, Laplace, and wavelet transforms directly because of the problems mentioned earlier. Some of the well‐known and thoroughly studied mathematical models of gene regulatory network of circadian rhythms are for Neurospora [6, 7, 8], Drosophila [8, 9, 10], and mammals [11, 12, 13, 14, 15]. Though these non‐linear ordinary differential equation (ODE) models are deterministic and are built based on the laws of mass action kinetics, they can also account for molecular noise [7, 16]. Popular Monte Carlo simulations like Gillespie's method [17] are employed to carry out stochastic simulations of gene regulatory network models of circadian rhythms. However, all the circadian properties from the model are obtained through numerical simulations under various conditions, since analytical solutions are difficult to obtain for coupled non‐linear ODE models. Circadian properties that are obtained through numerical simulations include a fitting model to the experimental time series to get the period and amplitude, to get the phase response curve (PRC) under pulse perturbations, and subjecting the model to various light–dark (LD) cycles to study entrainment properties. Therefore, analysis of simulated time series from the model through proper tools is highly important to get an insight into various properties of the circadian systems. Presently, to obtain the period of stochastic and deterministic time series from the model, various techniques like mean crossing method [18], Fourier [4], and wavelet transforms [1, 2, 19] were employed. Similarly, to construct PRCs, numerical methods like isochron and adjoint methods were used [20]. This indicates that diverse tools are used to extract different properties of circadian rhythms.

Interestingly, most of the important characteristic properties of the circadian rhythms like period, PRCs, entrainment properties, and robustness to molecular noise can be obtained from the instantaneous phases of the oscillations. Instead of using different techniques to obtain different properties of the circadian rhythms, in this work, we show that Hilbert transform (HT) can be used to extract most of the important circadian properties from the time series got from the circadian models. HT has been widely applied to diverse areas to analyse the time series of mechanical vibrations [21], to analyse data from geophysics [22], mass flow [23], and in bio‐medical research [24]. HT was also employed to construct PRC [25] and to extract phase information [3, 26, 27] from circadian data. Moreover, different groups used HT based methods to construct numerical PRC [28], to examine entrainment [29], and to study the synchronisation properties [30] of the oscillatory system. Besides circadian rhythms, HT was also used by Wang *et al.* [31] to study memory performance on visual and auditory data. In this work, we specifically show how HT can be used to determine period variations, construct PRC's, identify period sensitivity of the parameters to perturbations, and to get Arnold tongues to map the entrainment regions. We also compared our results of PRC, period sensitivity, and entrainment with the existing methods to show that our method is robust and versatile. We also propose a novel method (both model and numerics) in combination with HT to determine whether the circadian time series under forcing period conditions entrains completely (phase locking), partially (phase slips) or neither. We show that our method works well for both deterministic and stochastic time series under forcing conditions. We believe that this simple method will be useful to the experimentalists to quickly identify regions of entrainment to the external forcing period signals. To illustrate the use of HT in analysing circadian time series, we take as a test case the time series from the fruit fly model of circadian rhythms [10] and the time series from the experimental data [32] to demonstrate entrainment to different forcing cycles.

## 2 Materials and method

### 2.1 Circadian time series from the two‐variable model of fruit fly

We summarise first the Tyson *et al.* [10] model of fruit fly followed by the definition of HT. The gene regulatory model of fruit fly is based on the production of PER and TIM proteins by *per* and *tim* mRNA, respectively. These proteins are transported to the cytoplasm that may either undergo proteolysis or forms a stable product by heterodimerisation. The heterodimers in turn feedbacks and inhibits the *per* /*tim* transcription. However, Tyson *et al.* [10] proposed that the equilibrium between monomer and dimer results in the protection of dimers from the *double‐time gene* degradation and thereby increases the total concentration of monomer, dimer and its accumulation. This results in a double negative, positive feedback between monomer and dimer. Without considering PER and TIM separately, a three‐variable model was initially proposed with *M*, $P_{1}$ and $P_{2}$ that, respectively, are the mRNA, protein monomer and dimer. Then based on the assumption that the dimerisation reaction is fast, the model was reduced to two‐dimensional non‐linear ODEs that are reproduced below:

(1) $$\frac{dM}{dt}=\frac{v_{m}}{1+(P_{t}(1-q)/2P_{\text{crit}})){\;}^{2}}-k_{m}M$$

(2) $$\frac{dP_{t}}{dt}=v_{p}M-\frac{k_{P1}P_{t}q+k_{P2}p_{t}}{J_{P}+P_{t}}-k_{P3}P_{t}$$

(3) $$q=\frac{2}{1+\sqrt{1+8K_{\text{eq}}P_{t}}}$$

**Fig. 1:**
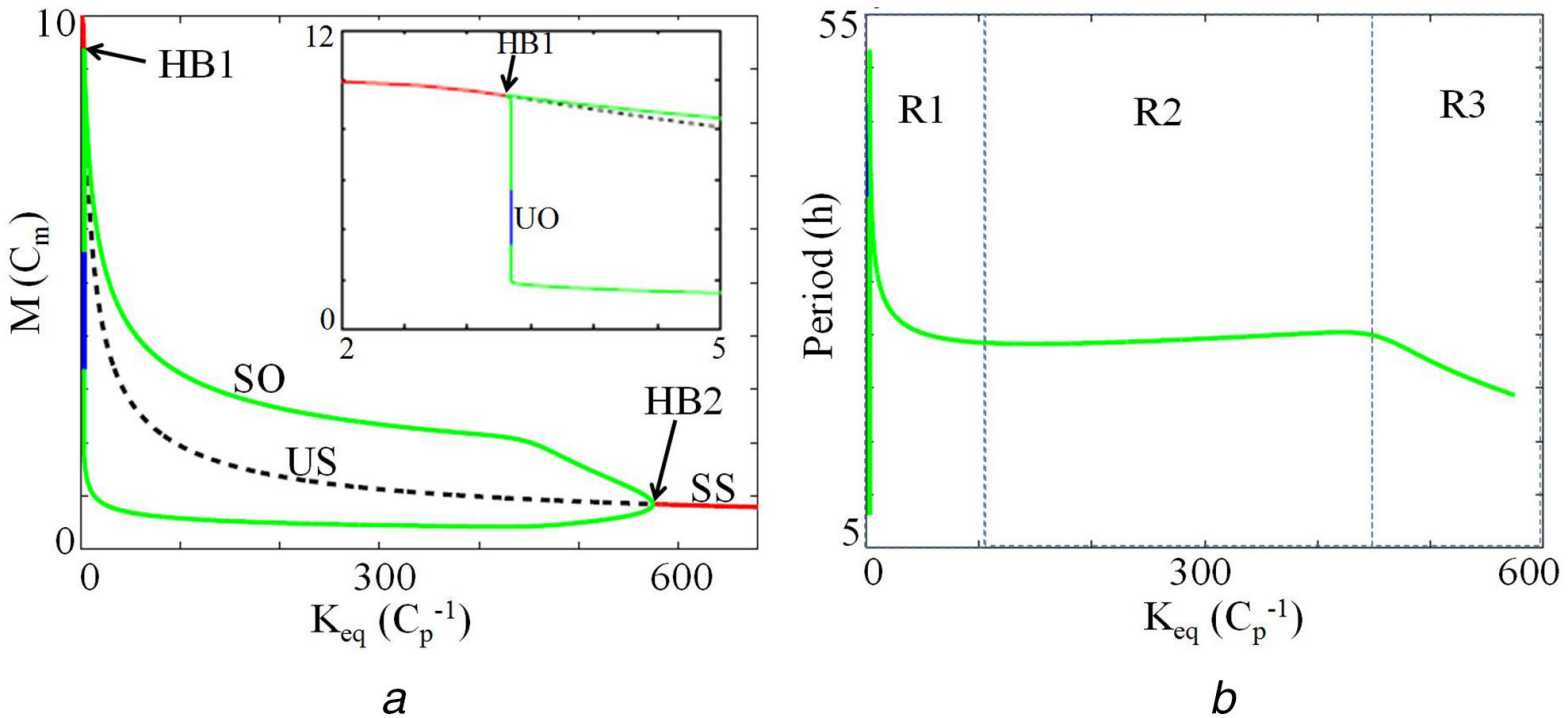
Codimension‐1 bifurcation diagram and bistability ***(a)*** For lower values of $K_{\text{eq}}$, the system is in the stable steady state (red lines, SS). As $K_{\text{eq}}$ increases oscillations appear via Hopf bifurcation (HB1). Black broken lines are the unstable steady state (US), blue line are unstable oscillation amplitude (UO), and green lines are stable oscillations amplitude (SO) of the variable *M*. Sustained oscillations disappeared via supercritical Hopf bifurcation (HB2), and the system enters the stable steady state. The inset shows the enlarged view of the bifurcation diagram for lower values of $K_{\text{eq}}$. Xppaut [33] is used for simulating the bifurcation diagram, ***(b)*** Period of oscillations as a function of $K_{\text{eq}}$. R1 and R3 are the regions where the period is highly sensitive to $K_{\text{eq}}$, whereas R2 is the region where the period is almost insensitive to $K_{\text{eq}}$. Simulation results are obtained by integrating (1) and (2) with parameter values $v_{m}=1\;C_{m}h^{-1}$, $k_{m}=0.1\;h^{-1}$, $v_{P}=0.5\;h^{-1}$, $k_{P1}=10\;C_{p}h^{-1}$, $k_{P2}=0.03\;C_{p}h^{-1}$, $k_{P3}=0.1\;h^{-1}$, $P_{\text{crit}}=0.1\;C_{p}$, $J_{P}=0.05\;C_{p}$. As used in [10], $C_{p}$ and $C_{m}$ are the characteristics concentrations for protein ($P_{t}$) and mRNA (*M*), respectively

In the equation, *M* is the mRNA and $P_{t}$ is the total protein, where $P_{t}=P_{1}+2P_{2}$. All the variables and parameters of the model are the same as in [10] unless specified otherwise. The parameter values are also provided in Fig. 1. The codimension‐1 bifurcation diagram for the mRNA (*M*) as a function of $K_{\text{eq}}$ is shown in Fig. 1. For lower values of $K_{\text{eq}}$, the system is in the stable steady state. As $K_{\text{eq}}$ increases, the stable steady state becomes unstable through a Hopf bifurcation (HB1) and generates oscillations. Further, an increase in $K_{\text{eq}}$ leads to a loss of periodic orbits through supercritical Hopf bifurcation (HB2) that has an unstable steady state surrounded by a stable limit cycle. We also show the variations of the period to the change in $K_{\text{eq}}$. As shown in Fig. 1 *b*, we identify three regimes in the bifurcation diagram. In regime‐I (R1), for low values of $K_{\text{eq}}$ (between 0 and 100 approx.), there is a rapid variation in the period that goes from 5 to 50 h, and this is the highly sensitive regime. In regime‐II (R2), between 100 and 450 of $K_{\text{eq}}$ values, the period is 24.2 h which is close to circadian. This wide regime is the most stable regime with the period being insensitive to parameter variations. In regime‐III (R3), ($K_{\text{eq}}$ between 450 and 580) the period is modestly sensitive to parameter variations. In this work, regime‐II (R2) is the region of our interest, and all the properties are studied for *K*_eq_  = 200. Using HT, we also perform period sensitivity analysis for the other parameters in regime‐II (R2) and regime‐I (R1), and we discuss this in detail in Section 4.

To assess the robustness of the model to molecular noise, we also perform a stochastic simulation of the two‐variable model. The deterministic model cannot describe the molecular fluctuations at the population levels, and therefore, we use the stochastic simulation algorithm (SSA) to determine the robustness of the model to noise through the HT method. We performed stochastic simulations according to Gillespie's method [17], and the results are discussed in Section 3. Gillespie's method, firstly, associates a probability to each of the reaction steps. Then at each time step, the algorithm randomly determines the occurrence of a particular reaction as well as the time interval to the next reaction step. The number of molecules of the different reacting species as well as the probabilities is updated at each time step. The transition probabilities of the reaction and the transitions are given in Table 1. The scaling parameter Ω is the system size (see [18, 34]) that can use to modulate the molecular noise; higher the Ω, less the molecular noise, and less the period variations. There are five reaction steps; each of them is associated with a probability, which depends on the deterministic kinetic parameters.

**Table 1:** Stochastic version of Tyson *et al.* [10] model. Deterministic to stochastic conversion is adapted from [7]

Number	Probability of reaction	Transition
1	$w_{1}=\frac{vm\Omega }{1+{\left(P_{t}(1-q)/2P_{\text{crit}}\Omega \right)}^{2}}$	M→M+1
2	$w_{2}=k_{m}M$	M→M−1
3	$w_{3}=v_{p}M$	$p_{t}\to P_{t}+1$
4	$w_{4}=\frac{P_{t}\Omega (k_{p1}q+k_{p2})}{Jp\Omega +P_{t}}$	$P_{t}\to P_{t}-1$
5	$w_{5}=k_{p3}$	$P_{t}\to P_{t}-1$
6	$q=\frac{2}{1+\sqrt{1+8(K_{eq}/\Omega )P_{t}}}$	—

The above 2D fruit fly model is also different from the existing gene regulatory circadian models of Drosophila, Neurospora, and mammals; (i) The model is 2D and is based on the positive feedback loop due to the dimerisation of PER protein. (ii) The model time series exhibits a sort of relaxation oscillations rather than smooth sinusoidal oscillations that are seen in all the existing models of circadian rhythms. (iii) The model exhibits both bistability and Hopf bifurcation for appropriate choice of parameters to explain the effect of light on mutants and normal phenotypes, respectively, whereas existing circadian models explains the behaviour of normal and mutant phenotypes only through Hopf bifurcations. (iv) The 2D model exhibits both Type‐I and II PRCs and the only simple gene regulatory model which we know of is the Goodwin's 3D model for Neurospora proposed by Ruoff *et al.* [6]. Finally, the model is also robust to molecular noise. This we show by performing a stochastic simulation by Gillespie's method [17] and by HT we find that the period distribution (variance) is close to the period (mean) of the fruit fly circadian oscillations (20–25 h).

### 2.2 Hilbert transform

HT converts the time‐domain signal x(t) to another time‐domain signal y(t) [35, 36]. HT of a signal x(t) can be considered as the convolution of signal x(t) and 1/πt [35]

(4) $$y(t)=\frac{1}{\pi }\int_{-\infty }^{\infty }\frac{x(\tau )}{t-\tau }dt$$

Simply, HT performs a −π/2 phase shift for every spectral component of x(t). A signal x(t) and its HT y(t) are mutually orthogonal and have the same autocorrelation and energy density spectrum [35, 36]. A complex time signal z(t) can be constructed using x(t) and y(t) which is called an analytic signal

(5) z(t)=x(t)+iy(t)

and in polar coordinates, z(t) is given as

(6) $$z(t)=a(t)\;e^{i\theta (t)}$$

with

(7) $$a(t)=[x^{2}(t)+y^{2}(t)]{\;}^{\left(1/2\right)},\;\theta (t)=\tan^{-1}\left(\frac{y(t)}{x(t)}\right)$$

a(t) is the envelope or amplitude of the analytic signal, θ(t) is the phase, and the time derivative of θ(t) provides the instantaneous frequency given as

(8) $$\text{Instantaneous}\;\text{frequency},f_{i}(t)=\frac{1}{2\pi }\frac{d\theta (t)}{dt}$$

Instantaneous frequency is a positive function of time that varies concerning the period of oscillation. The time average of the instantaneous frequency provides the average frequency in a signal spectrum [37]. HT is more suitable to analyse both stationary and non‐stationary data because of the following reasons:

(i) HT of a signal has the same frequency and amplitude of the original signal, which makes it a powerful tool for signal analysis.
(ii) HT is non‐local in time. Hence it is suitable for the computation of instantaneous characteristics of the signal, whereas Fourier transform and Laplace transform are local in time.
(iii) Unlike the wavelet transform, HT is versatile, which is adaptable to the different type of signals. However, in the wavelet transform analysis depends strongly on the proper choice of wavelet function that in turn depends on a specific signal.

We use *ode* 23*s* and *ode* 45 MATLAB® routines (Matlab 8.1, The MathWorks, Natick, MA) to get the time series from the 2D ODE model under various conditions. The Matlab programs are provided as supplementary materials.

## 3 HT method to determine the period of the circadian oscillator

One of the important characteristic features of circadian oscillator is its period. The period of oscillator determines the endogenous rhythm that is unique to the given species. Further, determining the period from the time series is an important aspect to understand and compare the dynamics of wild and mutant phenotypes behaviours of the circadian systems. However, oscillations observed in the experiments are noisy and extracting the period from a few, noisy, and some time the damping cycle is difficult. To capture this noisy oscillation, and it's period as seen in the experiments, both deterministic and stochastic simulations are carried out for the 2D model. Existing methods to calculate the period of noisy time series takes into account the time difference between the two consecutive peaks (trough), but these are not a very reliable method because many local maxima and minima will occur that lead to an inaccurate estimation of the period. To overcome this problem, Goldbeter and co‐workers [18], calculated the time difference between two consecutive upward crossing of mean levels of a suitable oscillatory variable. Another common method to determine the period is from the power spectrum of time series of any oscillatory variable. Though this gives the time‐invariant amplitude and frequency, it will not provide instantaneous frequency [38]. Therefore, we use the HT method to obtain the instantaneous frequency of an oscillator from the time series. Before we apply HT, we generate a time series by integrating the 2D model for the choice of kinetic parameters. We simulate both the deterministic (1) and (2) and stochastic versions (Table 1) of the model. For all the numerical analysis, we use the time series of the dynamical variable M(t), detrend it from its average, and convert it to an analytic signal of the form given in (5) by HT, from which we obtain the instantaneous phase (θ(t)) as in (6) and (7).

**Fig. 2:**
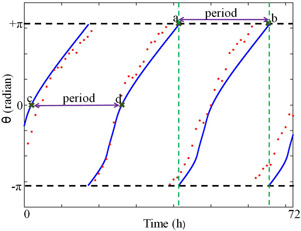
*Two different methods to determine the period from HT. Blue lines are the instantaneous phase of the deterministic model, and red dots are the instantaneous phase of the stochastic model. In the first method, points a* and *b are the two consecutive phase switching points, where the phase changes from* π *to* −π. *Note that at the phase switching points, the instantaneous phase of the stochastic model does not always reach to* π *or* −π. *In the second method, points c* and *d are the two consecutive zero crossing of the instantaneous phase*

We propose two different ways to determine the period numerically from the instantaneous phase of the time series. In the first method, we plot the instantaneous phase that changes from −π to π, i.e. the total phase change is 2π. Then we determine the time points where the absolute phase difference between two consecutive instantaneous phases, |θ(t)−θ(t+Δt)|≥1.5π (Fig. 2, points *a* and *b*). This time point indicates phase switch from −π to π and it provides the measure of one complete cycle of the oscillation (we choose 1.5π because, in stochastic simulation, it is not necessarily that the phase switch happens exactly from −π to π, see Fig. 2, red dots. Therefore, we arbitrarily took it as 1.5π, and this can be varied according to the noisy system). We repeat this for the whole time series and the time differences between two consecutive phase switching points (Fig. 2) give the period for one cycle. A similar approach has been employed previously for understanding the dynamics of brain signals [39]. In the second method, we extract the points where there is a positive zero crossing of the instantaneous phase (Fig. 2, points *c*, *d*). We then calculate the period by taking the time difference between two consecutive zero crossings. However, we use the phase‐switching method to determine the period in all our calculations. In supplementary, we describe the algorithm for HT‐based numerical method to calculate the period and the corresponding MATLAB programs.

**Fig. 3:**
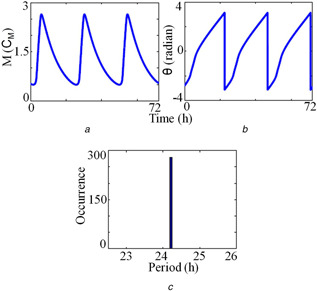
Period distribution of the deterministic time series ***(a)*** Time series of the variable *M*, ***(b)*** Instantaneous phase of the time series calculated via HT method, ***(c)*** Period distribution of the model. The endogenous period is close to 24.2 h as reported in the literature [10]. Simulation results are obtained by integrating (1) and (2) with $K_{\text{eq}}=200C_{p}^{-1}$. Rest of the parameter values are the same as in Fig. 1. As used in [10], $C_{p}$ and $C_{m}$ are the characteristics concentrations for protein ($P_{t}$) and mRNA (*M*), respectively

**Fig. 4:**
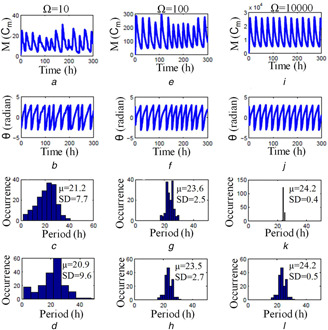
*Stochastic simulation, determination of instantaneous phase and period distribution of Tyson et al.* [10] *model using the HT method* ***(a)*** Time series of the variable *M* obtained from the stochastic simulation for Ω=10, ***(b)*** Instantaneous phase, ***(c)*** Period histogram calculated via mean crossing method [18], ***(d)*** Histogram of the period obtained via HT method. The distribution of the circadian period is over the range of 0–50 h. In all cases, the mean period and standard deviations for a single run are provided in the figure itself, and we calculated the period for the time series simulated for 2500 h. The average period is 19.13 h for ten simulations (not shown), and each simulation is carried over for 2500 h, ***(e)*** Time series for Ω=100, ***(f)*** Instantaneous phase, ***(g)*** Period histogram calculated via mean crossing method, ***(h)*** Period histogram calculated via HT. The distribution of the period is over the range of 18–30 h. The average period is 23.23 h for ten simulations (not shown), each over 2500 h, ***(i)*** Time series for the stochastic simulation for Ω=10,000, ***(j)*** Instantaneous phase, ***(k)*** Period histogram calculated via mean crossing method, ***(l)*** Period histogram calculated via HT. The average period is 24.14 h for ten trials, each trail simulated for over 2500 h. As Ω increases, the mean period obtained by stochastic simulation approaches to a deterministic value (24.2 h) and the standard deviation of the period distribution is very less. As used in [10], $C_{m}$ is the characteristics concentrations for mRNA (*M*)

The time series obtained from the deterministic model is shown in Fig. 3 *a* and the instantaneous period and the period distribution is shown in Figs. 3 *b* and *c*. HT is an infinite impulse response (IIR) filter, which requires computation on an infinite timescale. However, software package like Matlab implements finite impulse response (FIR) HT by applying window method [40]. This window method may distort the beginning and end of the data, which is called as the windowing effect [28]. To reduce the windowing effect, and to calculate the mean period, we drop the five oscillation cycles at the beginning and the end of the simulated time series. As reported in the literature [10], the mean period of the fruit fly oscillator obtained by HT method is 24.2 h. We then proceed to compute the period of the stochastic time series using the Gillespie method (Fig. 4). We use three different Ω values for stochastic simulation: Ω=10, Ω=100, and Ω=10,000. It is clear that the effect of molecular noise is more when Ω=10 and shows very large fluctuations in oscillations (Fig. 4 *a*). The time series also shows a large number of local maxima and minima in a single cycle, and the amplitude values take a very wide range. We perform the stochastic simulation for 2500 h to calculate the period distribution via an HT method for Ω=10 (Fig. 4 *d*), Ω=100 (Fig. 4 *h*) and Ω=10,000 (Fig. 4 *l*). Our results are on par with the mean crossing method [18] shown in Figs. 4 *c*, *g* and *k*. As in the case of any stochastic circadian model, the simulation of this 2D model also shows the robust circadian oscillations even when a fewer number of molecules are present. For Ω=10,000, the model shows the robust circadian oscillations, the period histogram is much narrower, and the mean period is close to the deterministic value.

In subsequent sections, we take both the deterministic and stochastic time series to study the characteristic properties of circadian rhythms using HT. We also use this time series to validate the novel method that we propose in the later section to determine the phase variations of forced circadian oscillator under different external forcing period conditions.

## 4 Influence of kinetic parameters on the period: period sensitivity from HT

A robust circadian system has to maintain the amplitude, period, and phase relationship between the molecular components regardless of the environmental variations and fluctuations. Besides robustness to molecular noise, another measure that provides information about the robustness/sensitivity of the model is the variations in the amplitude or period in response to parameter perturbations. The period of oscillations (τ) depends on the reaction rate parameters, and a small perturbation (Δp) in these parameters ($p_{j}$) may cause a large change in the period. Understanding period sensitivity provides insight into the functioning of the circadian system, and hence, helps to identify period sensitive parameters to modulate the period of the oscillator to simulate various phenotypes. Previous works have developed different methods to measure the period sensitivity of the circadian models [20, 41]. In [20], period sensitivity was determined from the rate of phase difference accumulated around each cycle, and we call this as the phase accumulation method. This method requires to solve the adjoint equation through backward integration, and this leads to instability and computational difficulty. To overcome this difficulty, we use HT to calculate the period sensitivity based on the finite difference method that is given below:

(9) $$\frac{\partial \tau }{\partial p_{j}}≃\frac{\tau (p+\Delta p)-\tau (p)}{\Delta p}$$

**Fig. 5:**
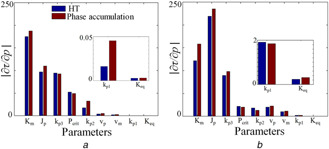
Comparison of HT based period sensitivity with phase accumulation method ***(a)*** Period sensitivity for WT ($K_{\text{eq}}=200$) calculated via HT method (blue) and the period sensitivity is calculated via the phase accumulation method as described in [20] (brown). $k_{p1}$, $K_{\text{eq}}$ are almost insensitive to the period of oscillations (inset), and this agrees with the published results [10, 20], ***(b)*** Blue indicates the period sensitivity calculated via HT for mutant ($K_{\text{eq}}=15$) (R1 in Fig. 1 *b*), and brown represents the period sensitivity calculated via phase accumulation method. Period sensitivity of $K_{\text{eq}}$ is higher for mutant than that of the wild type (inset)

We calculate the period of unperturbed (τ(p)) and perturbed (τ(p+Δp)) system by HT method. We rank‐order the parameters for wild‐type (WT) according to the absolute magnitude of sensitivity (Fig. 5 *a*), and the ranking matches with the reported results [20]. This ranking of the model parameter perhaps may help to redesign the whole model or may help to reduce the number of dynamical variables in the model that are insensitive. Top‐ranked sensitivity parameter, $k_{m}$ is the exponential degradation constant of mRNA (*M*). Our sensitivity analysis reveals that the model shows a period insensitivity to two crucial parameters, $K_{\text{eq}}$ and $k_{p1}$, and agrees with the reported results [10]. However, the sensitive parameters are present mostly in the mRNA (*M*) equation. We also perform the period sensitivity analysis when the $K_{\text{eq}}$ is low. As shown in Fig. 1 *b*, there are three regimes where R2 is the regime of wild‐type and R1 is the regime, where the period is high for a very low $K_{\text{eq}}$ value and this regime is taken as the mutant regime. We took $K_{\text{eq}}$ as 15 to simulate the period sensitivity of the mutant (Fig. 5 *b*). We find that for a very low $K_{\text{eq}}$ value, most of the parameters involved are in the production, degradation, and positive feedback loop of total protein $P_{t}$ ($J_{p}$, $v_{p}$, and $k_{p1}$) are more sensitive to variations in period than in the wild type. The period sensitivity of $K_{\text{eq}}$ is also higher (Fig. 5 *b*, inset), and this result agrees with the bifurcation diagram in Fig. 1 *b* that period change is higher for the mutant (R1) than in the wild‐type (R2). This indicates that positive feedback may be playing a strong role in influencing the period in R1 regime. Thus HT method captures the period sensitivity to variations in parameters of both wild and mutant types effectively.

## 5 Phase response curves

A plot of the perturbed phase against the phase shift for one circadian cycle due to the application of pulse stimulus provides the PRC. These curves explain the relationship between a small duration of the light stimulus applied at different phases in one cycle, and the corresponding phase shifts. PRC provides vital information about the range of entrainment of a circadian system, which in turn helps to understand the adaptation to external seasonal variations like long and short day cycles. PRC's are also categorised as Type‐1 and Type‐0 depending on the jumps in the phase shifts from delay to advance or vice‐versa [42]. PRC for various circadian systems under different conditions is well documented (e.g. Drosophila [43] and mammals [44]). To determine the goodness of the mathematical models of circadian rhythms, PRC is generated by light pulse stimulation, and its effect is compared with the experimental data. Different numerical methods have been previously developed to calculate the PRC [20, 33] and one of them use the adjoint method to construct PRC. In this section, we proposed HT‐based numerical method to the construct PRC of the 2D model of the fruit fly and compared our results with PRC got from the adjoint method [20]. For this, we first consider the circadian ODE model of the form

(10) $$x^{\prime }(t,p)=f(x(t,p)$$

Integrating the above equation gives the vector of state variable *X*:

(11) $$X(t,p)=\left\{x_{1},x_{2},\ldots ,x_{i},\ldots ,x_{n}\right\}$$

and p is the vector of parameters given as

(12) $$p=\left\{p_{1},p_{2},\ldots ,p_{j},\ldots ,p_{m}\right\}$$

$\tau_{0}$ is the free‐running period of the system. Discretising the time over one period gives

(13) $$t_{dt}=\left\{t_{1},t_{2},\ldots t_{k},\ldots t_{q}\right\}$$

where $t_{q}$ be the time at which phase is circadian time 24 (CT24).

Let *S* be the stimulus applied to perturb the system and it is given as

(14) $$S_{j}=\left\{\begin{array}{cc} p_{j}+\Delta p_{j}, & \text{for}\;\;t\in [t_{k},t_{k}+d]\\ p_{j},\;\; & \text{for}\;\;t\notin [t_{k},t_{k}+d] \end{array}\right.$$

where $\Delta p_{j}$ is a pulse given at the time $t_{k}$ for the time duration *d*. We compute the PRC for the parameter $p_{j}$

(15) $$\text{PR}C_{j}=\left\{\Delta \theta_{j1},\Delta \theta_{j2},\ldots ,\Delta \theta_{jk},\ldots ,\Delta \theta_{jq}\right\}$$

where $\Delta \theta^{jk}$ is the average value of phase differences between the perturbed and unperturbed phase of the state variable given below:

(16) $$\Delta \theta^{jk}=\frac{1}{N}\sum \theta_{\text{perturbed}}^{jk}-\theta_{\text{unperturbed}}$$

Here *N* is the total number of discrete points for which we calculate the Hilbert phases. $\theta_{\text{perturbed}}^{jk}$ is the phase of the perturbed system, where the parameter $p_{j}$ is perturbed at a time $t_{k}$ for the time duration *d* while $\theta_{\text{unperturbed}}$ is the phase of the unperturbed system. Positive and negative phase differences imply, respectively, phase lead and phase lag. We also describe below how to calculate the phase difference between the perturbed and unperturbed limit cycle oscillation using HT

(17) $$\theta_{\text{perturbed}}^{jk}=\tan^{-1}\left(\frac{X_{pH}^{jk}}{X_{p}^{jk}}\right)$$

(18) $$\theta_{\text{perturbed}}=\tan^{-1}\left(\frac{X_{Hu}}{X_{u}}\right)$$

where $X_{pH}^{jk}$ and $X_{Hu}$ are the HT of perturbed ($X_{p}^{jk}$) and unperturbed ($X_{u}$) state variables, respectively. Thus

(19) $$\Delta \theta^{jk}=\frac{1}{N}\sum \tan^{-1}\left[\frac{X_{pH}^{jk}X_{u}-X_{Hu}X_{p}^{jk}}{X_{p}^{jk}X_{u}+X_{pH}^{jk}X_{Hu}}\right]$$

We provide in the supplementary the Matlab program for the numerical computation of PRC.

**Fig. 6:**
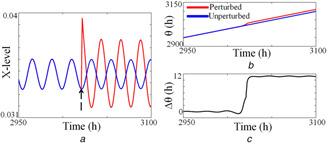
Illustration of phase difference obtained from the perturbed and unperturbed oscillator via the HT method ***(a)*** Unperturbed time series is shown as in blue line and perturbed time series as in the red line. The black broken arrow indicates the phase at which the perturbation is applied, ***(b)*** Instantaneous phases of perturbed and unperturbed time series calculated via HT, ***(c)*** The phase difference between the perturbed and unperturbed time series. Time series is obtained by integrating the ODE‐provided in [6], where the variable *X* represents the mRNA

**Fig. 7:**
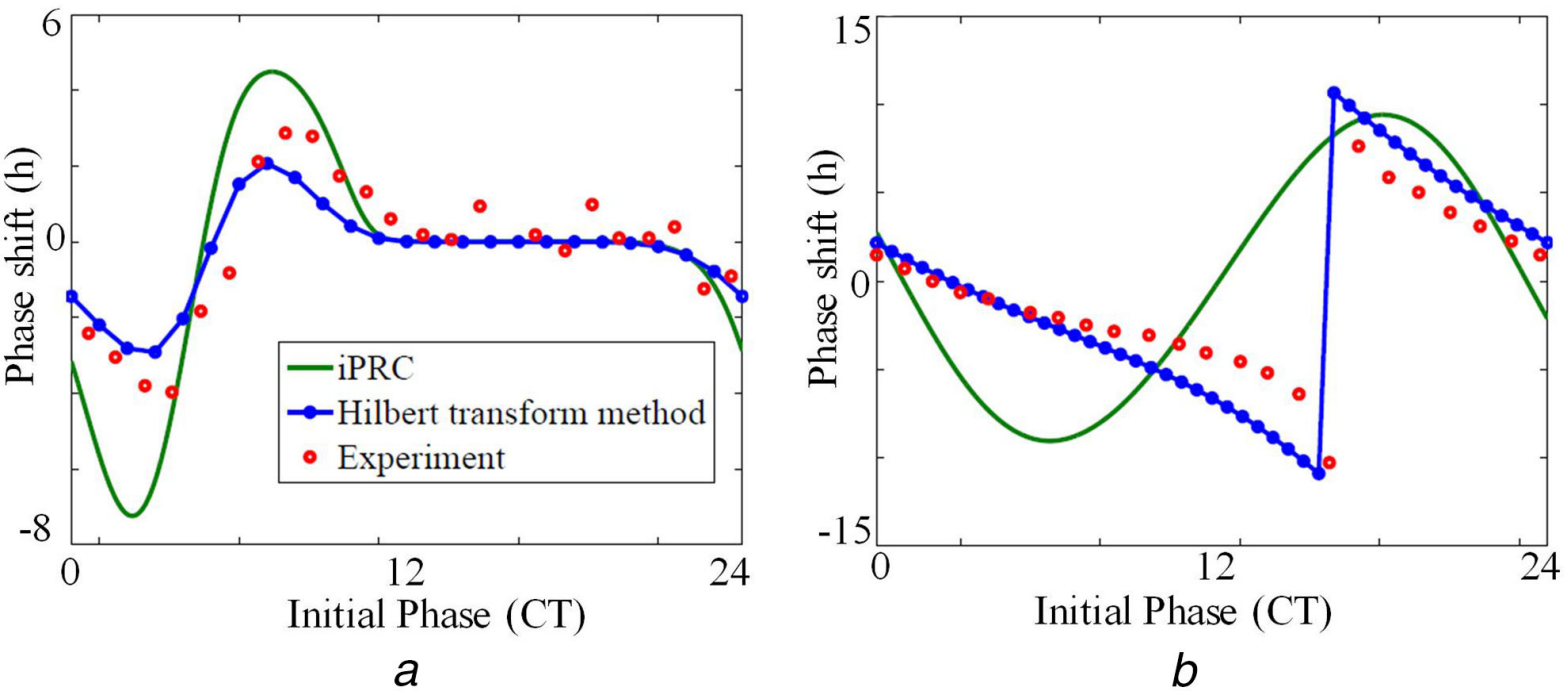
Comparison of PRCs by HT and iPRC methods for the Drosophila and Neurospora circadian models ***(a)*** PRC of the Drosophila is constructed from the circadian model described by the equations (1a)–(1j) in [8]. The experimental data of PRC was extracted from [44]. To simulate PRC, we set CT6 as the maximum of *per* mRNA. The light perturbation here takes the form of a 3 h long two‐fold increase in the parameter $V_{dT}$. PRC obtained via both iPRC (green line), and HT methods (blue line) are projected along with the experimental data (red circles). The *x* ‐axis is converted into circadian time (1CT(*h*) = 59.5 min), ***(b)*** PRC is constructed from the Neurospora circadian model described in [6], and the experimental data of PRC is also extracted from [6]. To simulate PRC, the maximum of *X*, the mRNA variable is set as CT4. PRC calculated via HT (blue line) and iPRC methods (green line) are projected along with the experimental data (red dots). The perturbation here takes the form of a 1 h long two‐fold increase in the parameter $k_{1}$ in the Goodwin model of Neurospora. The *x* ‐axis again converted to the circadian time (1CT(*h*) = 55.75 min). PRC's of both *Neurospora crassa* and Drosophila via HT method matches well with the experimental data in comparison to the iPRC method

**Fig. 8:**
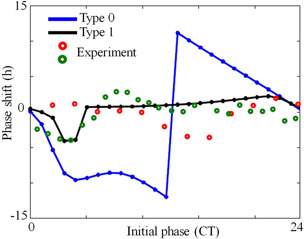
Type‐0 and type‐1 PRC of Tyson et al. model [10]. PRC obtained via HT method. For small amplitude perturbations, the model shows type‐1 PRC, and for high amplitude perturbation, type‐0 PRC is seen. Calculated PRC is matched with the published result (Fig. 5 a in [10]). For type‐1 PRC, $K_{eq}$ *is changed from 200 to 15 for 0.7 h, and for type‐0 PRC,* $K_{eq}$ is changed from 200 to 2 for 2 h. Experimental PRC is extracted from [43] (red circle) and [45] (green circle). Note the model published by Tyson et al., the PRC did not match well with the experimental PRC. So the simulated PRC from HT method also did not match with the experimental PRC but matched exactly with the model PRC simulated [10]

To determine how well HT method performs in simulating PRC of various species, besides getting the PRC for the fruit fly model [10], we also generate PRC of other well‐known circadian models of Drosophila [8], Neurospora [6], and compared the results with the experimental PRC. Fig. 6 shows how the parameter perturbation affects the time series (Fig. 6 *a*) and their phase (Fig. 6 *b*). The phase difference between the Hilbert phases of the perturbed and unperturbed time series is shown in Fig. 6 *c*. We specifically compared our method with the infinitesimal PRC (iPRC) method to capture the PRC's of Drosophila [8] and Neurospora [6] (see Fig. 7). The iPRC method requires to solve adjoint equations that are unstable and to get the solution requires backward integration that is difficult to program. Further, the PRC of Drosophila model obtained from the iPRC method is qualitatively very similar (Fig. 7 *a*, green line) but quantitatively incorrect. This method also works well only if the perturbation is very small [41], and for large perturbation, this method fails to predict PRC accurately. This method also fails to capture the Type‐0 PRC of Neurospora model (Fig. 7 *b*). HT‐based method, on the other hand, could reproduce quantitatively and qualitatively both Type‐0 and Type‐1 PRC's. Our method is efficient and accurate in comparison to the iPRC method, and this is because our algorithm computes the instantaneous phase of the oscillation for a large number of cycles and take its average. We also construct Type‐0 and Type‐1 PRC's of the 2D fruit fly model (Fig. 8), and it agrees well with the simulated results [10]. However, the model in [10] fails to reproduce the experimentally observed PRC [43, 45].

## 6 Mapping the entrainment region – Arnold tongue

**Fig. 9:**
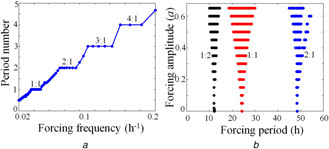
Devils staircase and Arnold's tongue ***(a)*** Devil's staircase. Period number as a function of forcing frequency. Some regions show a constant period number for a range of frequency, ***(b)*** Arnold's tongue. When the oscillator period is close to forcing signal period, entrainment occurs (1:1). Outside the entrainment region, the quasi‐periodic behaviour is observed. In 1:2 region, we observe for every one cycle, there is a two‐cycle forcing period, whereas in 2:1 region, for every two cycles, there is one cycle of forcing period. We obtain the Devil's staircase and Arnold's tongue by integrating (1) and (2). For LD cycle simulation, we use $K_{\text{eq}}=200(1-a)$ in the light phase, and $K_{\text{eq}}=200$ at the dark phase, where *a* it is proportional to the intensity of illumination [10]. To simulate Devil's staircase, we use a=0.5 with a duty cycle of 50% for the LD cycle. The period of the oscillator is calculated using the HT method. Arnold's tongue matches the reported result (Fig. 5 *b* in [10])

Entrainment happens when the frequency of a circadian oscillator is relatively close to the frequency of external forcing signal. The ratio of the period of the forced oscillator and the period of forcing signal is called the period number (rotation number). The plot of period number as a function of forcing signal frequency shows a staircase structure, known as the devil's staircase [46, 47]. We construct the devil's staircase from the model described in (1) and (2), and it is shown in Fig. 9 *a*. Some regions of the devil's staircase show constant period number for a range of frequency, which is the entrainment regions. Entrainment states can also be represented by Arnold tongues, which are the regions of period locking, where external forcing signals and the resulting forced oscillations of the circadian model have their periods in a specific ratio. In the entrainment region, the model can show n:m period locking. Here *m* and *n* are the number of forcing signal cycles and forced oscillation cycle, respectively. In Tyson *et al.* model [10], the forcing signal is LD cycle. In the LD cycle simulation, we use $K_{\text{eq}}=200(1-a)$ for the light phase and $K_{\text{eq}}=200$ for the dark phase. Here *a* is proportional to the intensity of illumination [10], and we take it as the forcing signal amplitude. To simulate Arnold's tongue, we use the LD cycle with 50% photoperiod (duty cycle), which denotes a 24 h forcing period with 12 h light phase and 12 h dark phase.

We construct the entrainment zone by first choosing appropriate forcing signal and model parameters. We then integrate the two variable ODE's and calculate the period of oscillation ($\tau_{\text{oscr}}$) by applying HT to the time series of the *M* variable. We compared this period with the external forcing period ($\tau_{E}$) given by $|m-(\tau_{\text{oscr}}/\tau_{E})|\leq \epsilon$, where ϵ is a small value to account for integration error. If the above inequality is satisfied, then it is said to be 1:m entrainment, where *m* is the number of forcing cycles. Similarly, we also determine the n:m entrainment, where we calculate *n* number of oscillations in *m* forcing cycle. The Arnold tongue for the 2D model (Fig. 9 *b*) is in good agreement with the reported one (Fig. 5 *b* in [10]). From the figure, it is clear that if the strength (amplitude) of the forcing signal is increased/decreased, then the entrainment region also expands/shrinks, a common characteristic of limit cycle oscillators. The entrainment region provides the parameter *a* for which 1:1 phase locking of entrainment and phase slips are possible. This information is used in the next section to validate the new method we propose in combination with HT for phase entrainments.

## 7 Phase equations

Phase equation describes the dynamics of the phase difference between free running oscillator subjected to an external forcing signal. Phase locking happens during entrainment, where the phase difference between forcing signal and forced oscillator phases should maintain a stable value. Phase equation can be utilised to determine the phase locking, and hence entrainment and phase slips. In the subsequent part of this section, we explain this in detail.

### 7.1 Derivation of the scaled phase equation

Entrainment for the deterministic and stochastic model of circadian rhythms have been studied and discussed in [48], and they studied the effect of molecular noise on phase locking and phase slip phenomena in detail. We provide below the equations that we use to determine the nature of entrainments like phase locking and phase slips from the time series data for a given forcing period.

We consider $\theta_{\mathrm{ext}}$ and $\theta_{\text{inst}}$, respectively, the instantaneous phases in radians of the external (forcing) and forced oscillations that governs the circadian oscillators. The phase difference is

(20) $$\Psi =\theta_{\mathrm{ext}}-\theta_{\text{inst}}$$

and the rate of change of this phase difference (radians/h) is given as

(21) $$\frac{d\Psi }{dt}=\frac{d\theta_{\mathrm{ext}}}{dt}-\frac{d\theta_{\text{inst}}}{dt}$$

(22) $$\frac{d\Psi }{dt}=\omega_{\mathrm{ext}}-\omega_{\text{inst}}$$

Note that we have taken the time in hours (h). To get the period, we take $\omega_{\mathrm{ext}}=(2\pi /T_{\mathrm{ext}})$, $\omega_{\text{inst}}=(2\pi /T_{\text{inst}})$ and we substitute this in the above equation to get

(23) $$\begin{array}{rl} \frac{d\Psi }{dt} & =\frac{2\pi }{T_{\mathrm{ext}}}-\frac{2\pi }{T_{\text{inst}}}.\\ \frac{d\Psi }{dt} & =2\pi \frac{T_{\text{inst}}-T_{\mathrm{ext}}}{T_{\mathrm{ext}}T_{\text{inst}}}. \end{array}$$

where $T_{\mathrm{ext}}$ and $T_{\text{inst}}$ are the period of forcing signal and forced oscillator, respectively. $T_{\mathrm{ext}}$ have a constant value over the period of time, whereas $T_{\text{inst}}$ is varied over time, and the procedure for calculating $T_{\text{inst}}(t)$ will discussed in Section 7.2. We scale (23) and reduce to dimensionless by taking $\psi =(\Psi /\Psi_{0})$ and $\tau =(t/T_{\mathrm{ext}})$ and the scaled equation is

(24) $$\frac{\Psi_{0}}{T_{\mathrm{ext}}}\frac{d\psi }{d\tau }=2\pi \frac{T_{\text{inst}}-T_{\mathrm{ext}}}{T_{\mathrm{ext}}T_{\text{inst}}}$$

(25) $$\frac{\Psi_{0}}{2\pi }\frac{d\psi }{d\tau }=\frac{T_{\text{inst}}-T_{\mathrm{ext}}}{T_{\text{inst}}}$$

If we take the constant $\Psi_{0}$ to be 2π radians then the above equation becomes

(26) $$\frac{d\psi }{d\tau }=\frac{T_{\text{inst}}(\tau )-T_{\mathrm{ext}}}{T_{\text{inst}}(\tau )}$$

This is the dimensionless phase equation we use to calculate the phase slip. To calculate the rate of phase slip per day ($d\psi_{d}/dt$), we modify the above equation as

(27) $$\frac{d\psi_{d}}{d\tau }=\left(\frac{T_{\mathrm{avg}}-T_{\mathrm{ext}}}{T_{\mathrm{avg}}}\right)\times 24$$

where $T_{\mathrm{avg}}$ is the average period of the forced oscillator, and it is given as $\left(1/n)\sum (T_{\text{inst}}\right)$ with *n* being the number of cycles. Note the difference between (26) and (27), and we use these two equations in the next section to calculate the region of phase locking and phase slips for the given parameter as well as the time series for the fruit fly model.

### 7.2 Numerics of the phase equation: phase locking and phase slip

**Fig. 10:**
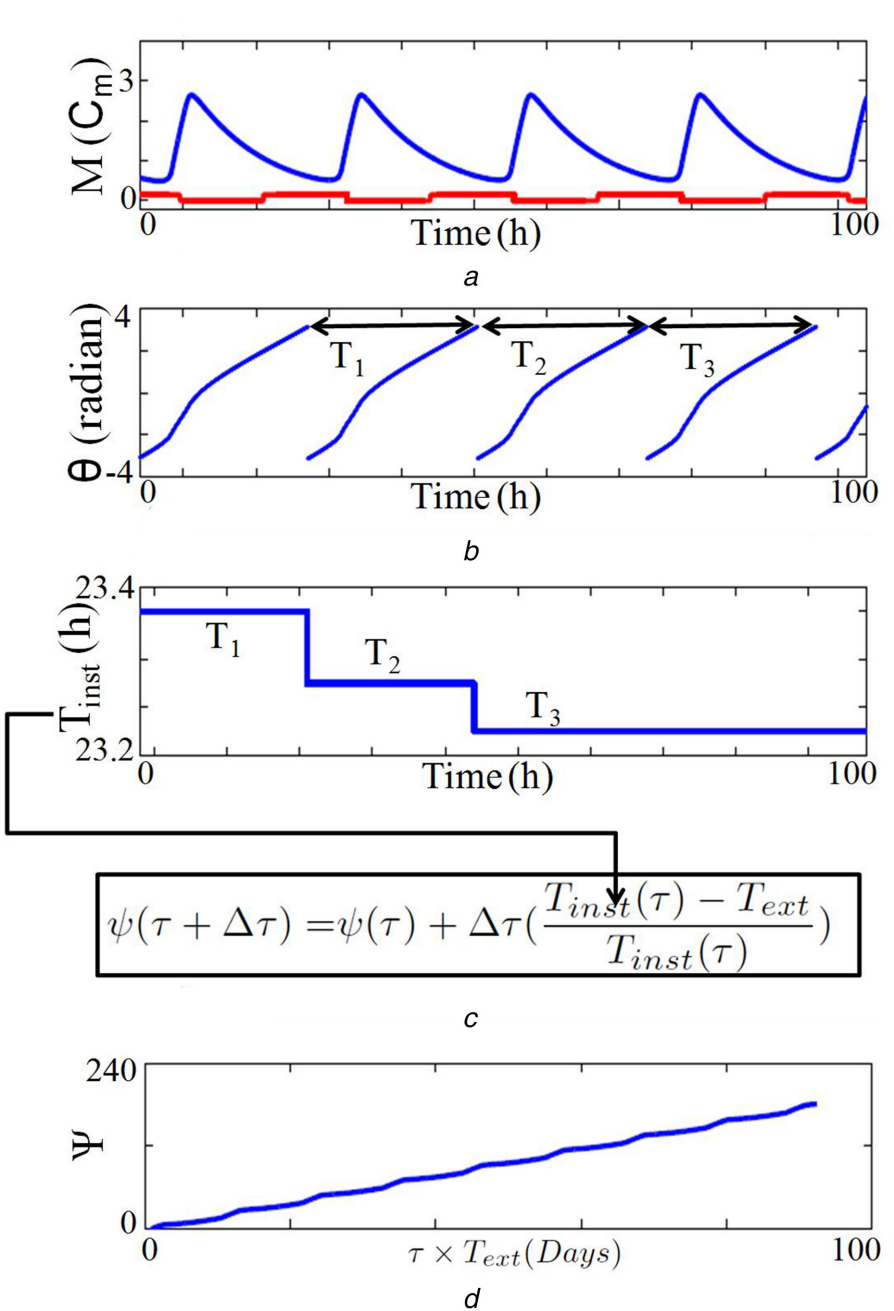
Calculation of phase slip ***(a)*** Time series is obtained from the model, ***(b)*** Instantaneous phase is calculated via HT. The period of each cycle is calculated as discussed in Section 3, and this period assigned as the instantaneous period ($T_{\text{inst}}(t)$) for one cycle ***(c)***, ***(d)*** This instantaneous period obtained is used for calculating the phase slip by routing it to (26). This equation is numerically integrated via Euler's method. As used in [10], $C_{m}$ is the characteristic concentration for mRNA (*M*)

To calculate phase slip, we compute the period for each cycle using HT and assume that the period is constant for one complete cycle of oscillation, we take this period as an instantaneous period ($T_{\text{inst}}$) for one complete cycle as shown in Fig. 10. We calculate phase slip by integrating (26) by Euler's method with an integrating step of Δτ, which is the same as the sampling time of the time series. We calculate the phase slip from the time series; we obtain from the fruit fly model [10] and the experimental data we got from the mice SCN culture [32] to illustrate the effectiveness of our novel method.

#### 7.2.1 Phase slip calculated for the time series obtained from the fruit fly model

**Fig. 11:**
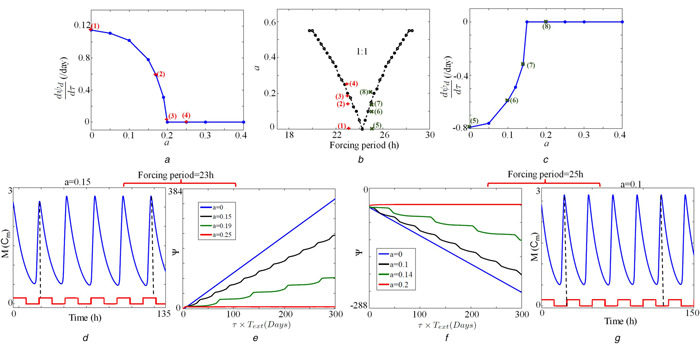
*Entrainment diagrams.* dψ/dt *versus a for the Tyson et al.* [10] *model with a forcing signal period of* ***(a)*** 23 h and, ***(c)*** 25 h. Calculated points are showing in the blue dots. Red ‘addition sign’ and green ‘multiplication sign’ are the corresponding points from the 1:1 Arnold's tongue ***(b)***, ***(d)*** Time series of the model with a forcing signal period of 23 h and amplitude value a=0.15. The model is not completely locked, and in each cycle, it shows different peaking time concerning external forcing signal. Black dotted line shows the peaking time of the variable *M*, ***(e)*** Phase slip calculated for a forcing signal with period 23 h and photoperiod 11.5 h for 300 days. When a=0, no locking occurs, and for non‐zero values of *a*, it shows the periodic tendency for locking, ***(f)*** Phase slip calculated for a forcing signal with period 25 h and photoperiod 12.5 h, ***(g)*** Time series of the model with a forcing signal period of 25 h and amplitude value a=0.1. As used in [10], $C_{m}$ is the characteristic concentration for mRNA (*M*)

We use two forcing periods 23 and 25 h to illustrate phase locking and slips in the fruit fly model [10]. When forcing amplitude a=0, the model oscillates with a free running period of 24.2 h. A phase slip of 1.2 h is obtained if the forcing period $T_{\mathrm{ext}}$ is 23 h and according to (27), the rate of phase slip per day (24 h) is 1.19 h. We also calculate the rate of phase slip per day for various values of *a* with a period forcing of 23 h and plot of $d\psi_{d}/dt$ versus *a* is shown in Fig. 11 *a*. There is a critical value of *a* below which the phase slip occurs, and above which forced oscillator is completely phase locked to the applied external signal. We also repeat this to the forcing signal with a period of 25 h. When a=0, phase slip occurs, and it is −0.8 h and the rate of phase slip per day is −0.79 h. We again obtain the rate of phase slip per day for different values *a* with forcing period 25 h and the plot of $d\psi_{d}/dt$ versus *a* is shown in Fig. 11 *c*.

We also numerically integrate (26) to calculate the phase slip and locking for different *a* values with two different forcing signals of period 23 and 25 h. The results are shown in Figs. 11 *e* and *f*, respectively, for various *a* values. We specifically select four different *a* values, three from the non‐entrainment region, and one from the entrainment region as shown in Fig. 11 *b* (red ‘addition sign’ and green ‘multiplication sign’). In Fig. 11 *e*, the period of the forcing signal is 23 h, when a=0 (without forcing signal), slip progress at a constant rate (blue line). When a≠0, the system exhibits the periodic tendency for locking (black and green lines). When a=0.2, the system is completely phase locked ((dψ/dt)=0, red line). Corresponding time series for a=0.15 are shown in Fig. 11 *d*. We observe similar results for the forcing period of 25 h (Figs. 11 *f* and *g*).

**Fig. 12:**
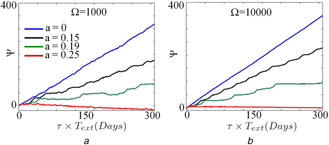
Effect of molecular noise on entrainment. The effect of molecular noise on the stochastic version of Tyson et al. model with ***(a)*** Ω=1000 and, ***(b)*** Ω=10,000. In all simulations, the period of forcing signal is 23 h

To see the effect of molecular noise on entrainment, we use the stochastic time series of the Tyson *et al.* model with a forcing period of 23 h and calculate the phase slip using (26). The results are shown in Fig. 12. When Ω=1000 and Ω=10,000, entrainment is seen for the higher values of *a* (Figs. 12 *a* and *b*). For Ω=10,000 (Fig. 12 *b*), the phase slip that we obtained from the stochastic simulations matches well with the deterministic simulation (Fig. 11 *e*). This indicates that the model is robust to noise and its entrainment property to the external forcing signal is similar to that of the deterministic model provided that Ω is large enough.

## 8 Validation of the phase equation: identification of phase‐locked regime in the entrained experimental time series of SCN under different forced temperature cycles

**Fig. 13:**
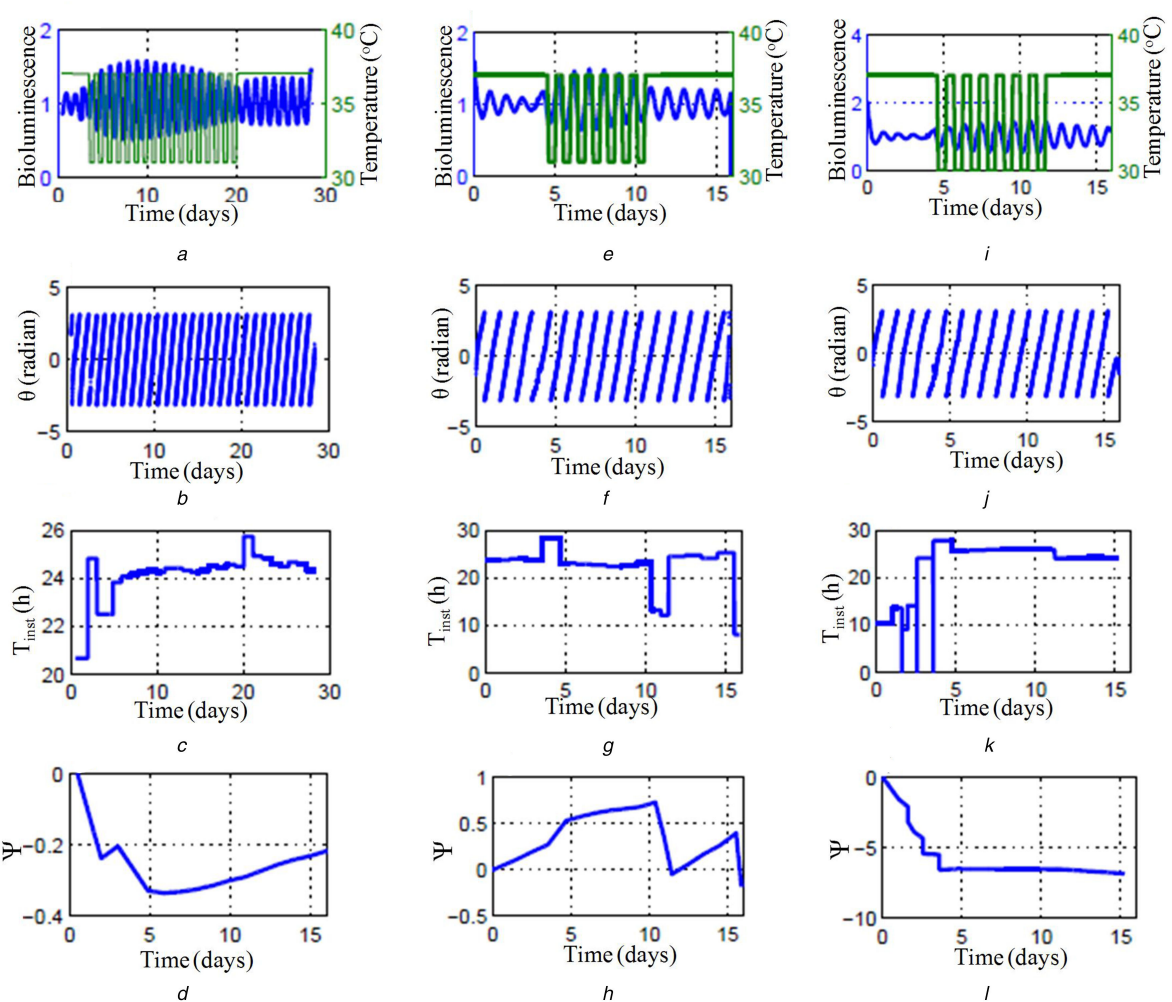
Entrainment of experimental SCN circadian time series to different forced external temperature cycles ***(a)***, ***(e)***, ***(i)*** Time series (blue line) of the bioluminescence signal from PER2::LUC mouse SCN slice [32]. Green lines indicate the various temperature levels during the days. Initially, the temperature kept at a constant level, and after a few days it drops to some lower level periodically with a forcing period ($T_{\mathrm{ext}}$) of ***(a)*** 24 h, ***(e)*** 22 h and ***(i)*** 26 h, ***(b)***, ***(j)***, and ***(f)*** represent the instantaneous Hilbert phase of the time series shown in ***(a)***, ***(e)***, ***(i)***, respectively. ***(c)***, ***(g)***, ***(k)*** $T_{\text{inst}}$ is calculated as described in Fig. 10. Calculated phase slip according to (26) for the time series concerning the forcing signal as shown in ***(a)***, ***(e)***, ***(i)*** are provided in ***(d)***, ***(h)***, ***(l)***, respectively

In this section, we validate HT‐based analysis of entrainment property to the experimentally collected circadian data. Bordyugov *et al.* [32] investigated the entrainment properties of mouse SCN slice time‐series data to the externally forced temperature cycles. They recorded the time series of bioluminescence signal from PER2::LUC mouse SCN slice under a different period of temperature cycle (forcing signal). Here, we analyse this SCN experimental data (Figs. 13 *a*, *e* and *i*) with our phase equation model to determine the entrainment of PER2 bioluminescence to the temperature cycle. For this, we first calculate the Hilbert phase of the detrended data (Figs. 13 *b*, *f* and *j*), and using this phase information; we calculate the instantaneous period ($T_{\text{inst}}$) as discussed in Section 7.2. The results are shown Figs. 13 *c*, *g* and *k*. The $T_{\text{inst}}$ obtained is routed to (26) to calculate the phase locking regime (Figs. 13 *d*, *h* and *l*). From HT‐based numerics, it is clear that PER2 entrains to the temperature cycles, and in the entrained region, temperature cycle and the SCN PER2 data have the same period (Figs. 13 *c*, *g* and *k*). Also, during the entrainment period, phase slip calculated through (26) is constant (Figs. 13 *d*, *h* and *l*). These results are in good agreement with the findings that SCN entrains to the temperature cycle for different periods of 22, 24, and 26 h, given the temperature variation is strong enough (6–8^°^ C temperature variation) [32].

## 9 Discussion and conclusion

In this work, we introduced the HT‐based method to analyse the time series of circadian models. HT is a powerful method that accurately determined the instantaneous phase and frequency, and from this information, we showed that the information about the period distribution of noisy time series, PRC's, Arnold tongue, and phase slip phenomena could effectively compute. In the presence of noise, obtaining frequency information from the simulated (stochastic) data is more challenging and hence it is difficult to calculate the period. However, we showed in Section 3 that HT can be utilised to calculate the period distribution of noisy time series obtained from stochastic simulation, and the estimated periods matched well with the published results. Further, the period of the oscillations depends on the model parameters, and the period sensitivity analysis helps to identify the parametric dependencies on the oscillatory period of the model. We showed in Section 4 that the period sensitivity using the HT method provides excellent results that are in agreement with the published results [20, 10].

PRC is a powerful tool to analyse the performance of the circadian model. Previously, experimental PRC of the SCN clock cell under temperature perturbation was constructed using HT [25]. Also, HT has been applied to construct numerical PRC of the oscillatory system [28]. However the amplitude information was utilised to calculate the phase difference. Unlike previous methods, with a detailed mathematical explanation, we directly utilised the phase information for constructing PRC in Section 5. Further, based on our method we constructed both Type‐0 and Type‐1 PRC's of various circadian models under light perturbation that agreed very well with the published results [6, 8, 10].

Entrainment property tells the ability of a circadian model to adapt to the day length variation of the environment. We applied the HT method to determine the period mismatch between circadian model and the external forcing signal by constructing Arnold's tongue in Section 6. We successfully reproduced the wedge‐like shaped region for 1:1, 1:2, and 2:1 entrainment regions for Drosophila model [10]. A phase slip phenomenon is another method to examine the entrainment property. Previously, in [29] the amount of entrainment is quantified using HT method. However, their method does not provide information about at which time entrainment (phase locking) or non‐entrainment (phase slip) occur. In this work, we proposed a simple model and numeric in Section 7 to determine the entrainment properties of the system under the external LD forcing periods. Importantly, we showed that phase locking and phase slips can be obtained from the time series data using the HT method. We specifically compared the results obtained from the HT method with the experimental data [32] and the results were found to agree very well. Importantly, we believe that this method can be used directly to the forced experimental data with known forcing periods to determine the occurrence of entrainment or not. In summary, we showed here that the HT‐based time‐series analysis of circadian data can be used to determine most of the circadian properties like period distribution, period sensitivity, PRC, and entrainment.

## Supporting information

Supplementary Data

Supplementary Data

Supplementary Data

Supplementary Data
