# Supplementary material for: Hilbert transform‐based time‐series analysis of the circadian gene regulatory network
Source: IET Syst Biol. 2019 Aug 1;13(4):159–68. doi: 10.1049/iet-syb.2018.5088 (PMC8687344; doi:10.1049/iet-syb.2018.5088)
Supplement: Supplementary file 1 — Supplementary Data [file SYB2-13-159-s004.pdf]

S1 Table: Algorithm

Algorithm for calculating period of oscillation, Arnold's tongue, PRC, and phase slip via HT method.

Algorithm	comments	Matlab functions
**Instantaneous frequency and period calculation**		
**Input**:$x'(t) = f(x(t), p)$	Input the circadian model	
$x(t) = \int f(x, p)$	Integrate the model for time series	$ode23s$
$x_d(t) = x(t) - \bar{x}(t)$	Substract the average number from the signal	$detrend$
$Z(t) = x_d(t) + iHT(x_d(t))$	Analytic signal generation	$hilbert(x_d)$
$\theta = tan^{-1}(imag(Z(t)/Real(Z(t))$	Find the Instantaneous phase	$angle(Z(t))$
if $\|\theta(t) - \theta(t+\Delta t)\| \geq 1.5\pi$		
$t_{sw}(end+1) = t$	store the phase switching time	
end		
$\tau = t_{sw}(i) - t_{sw}(i-1)$	Take the time difference between consecutive phase switching points	
**Arnold's tongue**		
**Input**: PP	Fix the photo period of forcing signal	
**Input**: $\tau = [\tau_1, \tau_2, ...\tau_{N1}]$	Forcing signal period	
**Input**: $A = [A_1, A_2, ...A_{N2}]$	Forcing signal Amplitude	
**Initialize**: $\tau_{E1} = []; A_{E1} = []$	Array for storing period and amplitude for 1:1 entrainment	
**Initialize**: $\tau_{E2} = []; A_{E2} = []$	Array for storing period and amplitude for 2:1 entrainment	
**Initialize**: $\tau_{E3} = []; A_{E3} = []$	Array for storing period and amplitude for 1:2 entrainment	
$for\ i = 1 : N_1$		
$for\ j = 1 : N_2$		
$x(t) = \int f(x, p, \tau_i, A_j, PP)$	Integrate the model for time series x	$ode23s$
Compute τ_x	Compute the period of the oscillator using HT method	
$If\ \|1 - \frac{\tau_x}{\tau_i}\| \leq \varepsilon$	Condition for 1:1 entrainment	
$\tau_{E1}(end+1) = \tau_i; A_{E1}(end+1) = A_j$	store the forcing period and amplitude of 1:1 entrainment	
$elseIf\ \|2 - \frac{\tau_x}{\tau_i}\| \leq \varepsilon$	Condition for 2:1 entrainment	
$\tau_{E2}(end+1) = \tau_i; A_{E2}(end+1) = A_j$	store the forcing period and amplitude of 2:1 entrainment	
$elseIf\ \|0.5 - \frac{\tau_x}{\tau_i}\| \leq \varepsilon$	Condition for 1:2 entrainment	
$\tau_{E3}(end+1) = \tau_i; A_{E3}(end+1) = A_j$	store the forcing period and amplitude of 1:2 entrainment	
$end\ if$		
$end\ for$		
$end\ for$		
**PRC**		
$x_u(t) = \int f(x, p, L_{tk} = 0)$	Generate an unperturbed limit cycle	
compute Z_1	Construct the analytic signal of unperturbed detrand data	$hibert(detrend(x_u))$
$\theta_1 = tan^{-1}(imag(Z(t)/Real(Z(t))$	Compute the unwraped phase of unperturbed signal	$unwrap(angle(Z1))$
$x_p(t) = \int f(x, p, L_{tk} \neq 0)$	Generate the perturbed limit cycle	
compute Z_2	Construct the analytic signal of perturbed detrend data	$hibert(detrend(x_p))$
$\theta_2 = tan^{-1}(imag(Z_2(t)/Real(Z_2(t))$	Compute the wraped phase of unperturbed signal	$unwrap(angle(Z2))$
$\Delta\theta_i(h) = \frac{period}{2\pi}(\theta_1 - \theta_2)$	Compute the instantaneous phase difference	
$PRC_j(t_k) = \frac{1}{N}\sum_{i=1}^{N}\theta_i$	Take the average of instantaneous phase differences. t_k	$mean(\Delta\theta_i)$
**Phase slip**		
**Input**: τ_E	Fix the period of forcing signal	
$for\ i = 1 : length(t_{sw})$		
$If\ t \geq t_{sw}(i) \& t \leq t_{sw}(i+1)$	Calculate the period of each cycle via HT method	
$T_{inst}(t) = t_{sw}(i+1) - t_{sw}(i)$	Assign the period to each cycle as instantaneous period	
$end\ if$		
$end\ for$		
$for\ i = 1 : length(T_{inst})$		
$\psi(i+1) = \psi(i) + d\tau(\frac{T_{inst}(i) - T_{ext}}{T_{inst}(i)})$	Calculate phase slip via Euler's method	
$end\ for$		
